# Druggability of Voltage-Gated Sodium Channels—Exploring Old and New Drug Receptor Sites

**DOI:** 10.3389/fphar.2022.858348

**Published:** 2022-03-17

**Authors:** Goragot Wisedchaisri, Tamer M. Gamal El-Din

**Affiliations:** Department of Pharmacology, University of Washington, Seattle, WA, United States

**Keywords:** ion channels, drug receptors, neurotoxins, sodium channel druggability, sodium channel pharmacology, drug development, gating-modifier toxins, fenestration

## Abstract

Voltage-gated ion channels are important drug targets because they play crucial physiological roles in both excitable and non-excitable cells. About 15% of clinical drugs used for treating human diseases target ion channels. However, most of these drugs do not provide sufficient specificity to a single subtype of the channels and their off-target side effects can be serious and sometimes fatal. Recent advancements in imaging techniques have enabled us for the first time to visualize unique and hidden parts of voltage-gated sodium channels in different structural conformations, and to develop drugs that further target a selected functional state in each channel subtype with the potential for high precision and low toxicity. In this review we describe the druggability of voltage-gated sodium channels in distinct functional states, which could potentially be used to selectively target the channels. We review classical drug receptors in the channels that have recently been structurally characterized by cryo-electron microscopy with natural neurotoxins and clinical drugs. We further examine recent drug discoveries for voltage-gated sodium channels and discuss opportunities to use distinct, state-dependent receptor sites in the voltage sensors as unique drug targets. Finally, we explore potential new receptor sites that are currently unknown for sodium channels but may be valuable for future drug discovery. The advancement presented here will help pave the way for drug development that selectively targets voltage-gated sodium channels.

## Introduction

### Overview of the Voltage-Gated Sodium Channel Family

Voltage-gated sodium channels (Na_V_) are targets of numerous clinical drugs that are used for treating diseases related to neural disorders (seizures and neuropathic pain), cardiac arrhythmia, and muscle illnesses. They are also the target for a class of drugs called local anesthetics (LA), which have been used for local and regional anesthesia (Catterall et al., 2020a). This is due to the critical role of these channels in generating and propagating electrical signals known as action potentials in the nervous system, the heart, and the skeletal muscles (Hille, 2001). Na_V_ channels are transmembrane proteins that are located in the plasma membrane of excitable cells. They selectively conduct sodium ions (Na^+^) from the extracellular fluid into the cell which results in changes in electrochemical gradients near the cell membrane and thus a depolarization of the membrane potential. Sodium conductivity through Na_V_ channels occurs when the channel is open and is tightly regulated by the membrane potential. Changes in the membrane potential trigger Na_V_ channel opening to initiate an action potential, which is subsequently propagated through the high density of Na_V_ channels in the membrane and leads to vital physiological processes, including neuronal communication and muscle contraction (Hille, 1986).

**FIGURE 1:**
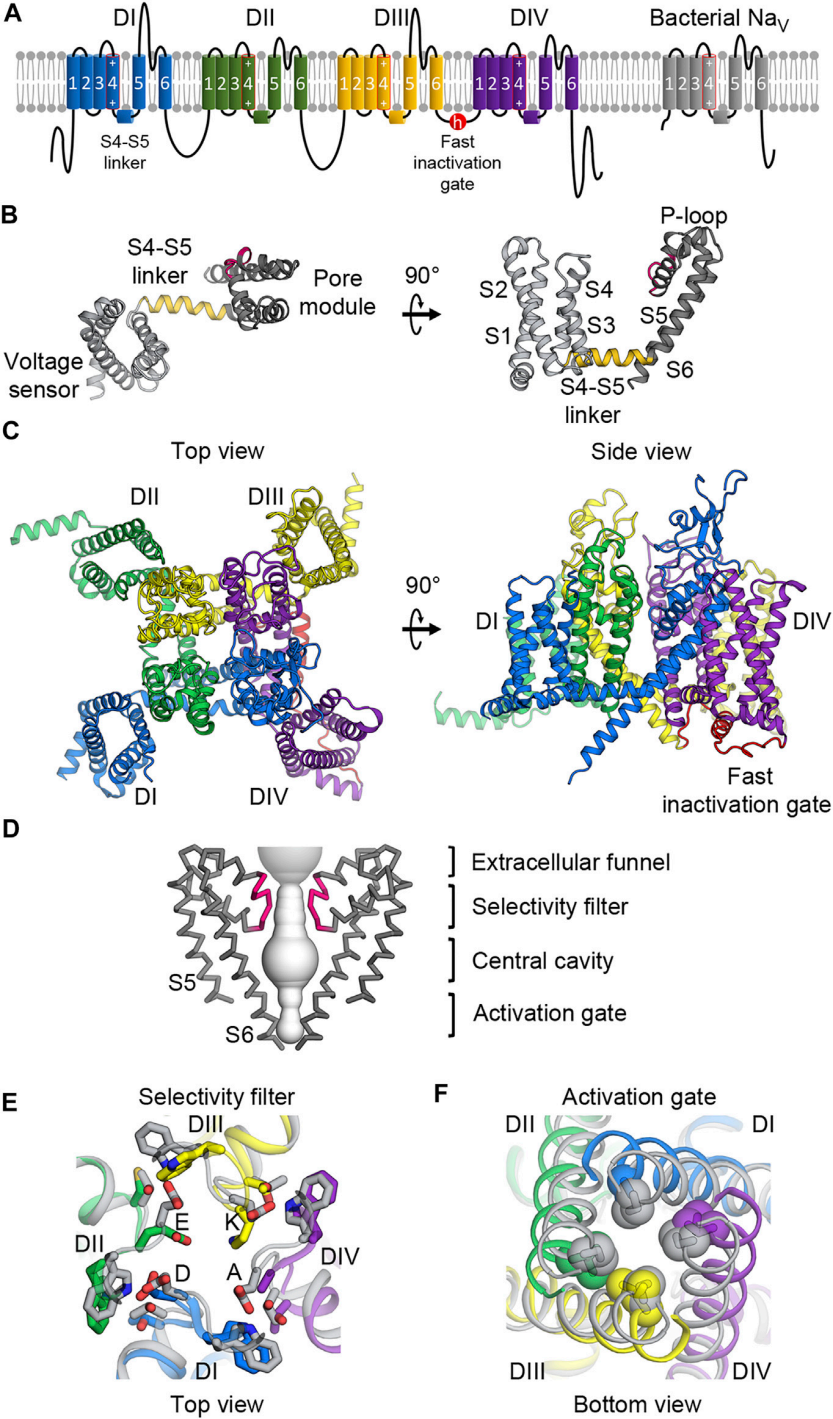
Molecular architecture of Na_V_ channels. **(A)** Topology diagram of mammalian and bacterial Na_V_ channels in the lipid membrane. Left, mammalian Na_V_ channels comprise four domains (DI—blue; DII—green; DIII—yellow; DIV—purple) with the fast inactivation gate (red) in the DIII-DIV loop. Right, bacterial Na_V_ channels contain one domain and lacks the fast inactivation gate. The S4 segments include an array of positive gating charge arginines. **(B)** Structure of the core Na_V_ channel domain from bacterial Na_V_Ab. The 6-TM domain consists of the voltage sensing domain (S1 to S4, light gray) and the pore module (S4 to S5, dark gray) connected by the S4-S5 linker (gold). Between the S5 and S6 segments is the P-loop including the selectivity filter (pink). **(C)** Structure of the mammalian Na_V_ α-subunit. Four domains colored as in **(A)** form a domain-swapped tertiary structure with the pore modules lining the pore architecture at the center, and the VSDs in the periphery.**(D)** Molecular architecture of the pore. The pore modules form a sodium permeable tunnel (light gray volume). For clarity, only two in-plane domains are illustrated. From top to bottom: the extracellular funnel attracts and concentrates Na^+^ ions; the selectivity filter (pink) selects for passages of hydrated Na^+^ ions; the central cavity contains hydrophobic inner surface; the activation gate formed by the S6 segments provides an iris-like exit. **(E)** Extracellular view of the selectivity filter superimposed between mammalian and bacterial Na_V_ channels. Mammalian Na_V_ channels colored as in **(A)** contain the DEKA motif to which each domain contributes an amino acid while the bacterial selectivity filter (gray) is symmetric with glutamate residues forming a high-field-strength site. **(F)** Intracellular view of the activation gate superimposed between mammalian and bacterial Na_V_ channels. The S6 segments interlace with hydrophobic residues sealing the exit when the channel is closed, and dilating when the channel is open.

Functional Na_V_ channels are composed of a pore-forming α-subunit (Figure 1A) and one or two of auxiliary β-subunits. There are nine Na_V_ α-subunit genes that encode the mammalian Na_V_ channel subtypes Na_V_1.1 to Na_V_1.9 (*scn1a* to *scn5a*, and *scn8a* to *scn11a*, respectively). They show 50–80% sequence identity and have distinct tissue distributions (Catterall et al., 2020a). Na_V_1.1, 1.2, 1.3, and 1.6 are found predominantly in neurons of the central nervous system. Na_V_1.4 is expressed in the skeletal muscles while Na_V_1.5 is distributed in cardiac muscle tissues. Na_V_1.7, 1.8, and 1.9 are located largely in the neurons of peripheral nervous system. There are four auxiliary β-subunits (β1 to β4) encoded by the *scn1b* to *scn4b* genes in mammals that are expressed ubiquitously and form complexes with the pore-forming α-subunit to regulate the function of the holo-channels (Calhoun and Isom, 2014). The combination of different β-subunits with the α-subunit gives rise to functional variations and complexity in channel modulation.

## State-Dependent Structure and Function of VOLTAGE‐GATED SODIUM Channels as Keys to Druggability

Ion channels function through a cycle of different structural conformations, or states, to which certain drugs and toxins can bind in a state-dependent manner. To understand and rationally design drugs that target a specific state of an Na_V_ channel, it is important to recognize how the channel functions at the biophysical level. This knowledge has been gathered using different functional techniques including electrophysiology over several decades before the era of structural biology.

Ions have long been known as the main cause of excitability of muscles and nerves. Sidney Ringer showed the importance of ions for the contractility of the heart in the 1880s (Hille, 2001). Julius Bernstein described correctly that the membrane polarizability results from the fact that the plasma membrane is permeable to K^+^ ions at the resting state and to other positive ions at the activated state (Hille, 2001). He predicted that the membrane resting potential could be calculated using the Nernst equation. However, the examination of the real value for the resting membrane potential did not happen until the 1940s when advanced microelectrode recording techniques were developed (Bretag, 2017). Our general understanding of how Na_V_ channels operate came from functional studies dated back to early works by Alan L. Hodgkin and Andrew F. Huxley in the 1950s (Hodgkin and Huxley, 1952d; Hodgkin and Huxley, 1952e). They described the sodium current that initiates the nerve action potential and established the molecular framework for voltage-dependent activation, fast inactivation, and sodium selectivity. Through their model, they envisioned the existence of three gating elements (which they called the **m** particles) that must be activated to gate the channel open. They also predicted the fourth gating particle, which they called the **h** particle, responsible for inactivating the channel. Much of the progress in structure-function relationships of Na_V_ channels came from subsequent decades of electrophysiological, biochemical, and mutagenesis studies (For comprehensive reviews, see (Ahern et al., 2016; Catterall, 2012; Hille, 2021)) that have recently been further solidified by structural studies of bacterial and mammalian Na_V_ channels in various conformations. Na_V_Ab from *Arcobacter butzleri* was the first Na_V_ structure to be solved by X-ray crystallography (Figure 1B) (Payandeh et al., 2011) and was soon followed by bacterial Na_V_ structures in multiple conformations (Payandeh et al., 2011; Zhang X. et al., 2012; McCusker et al., 2012; Payandeh et al., 2012; Sula et al., 2017). When mammalian Na_V_ structures finally became available, thanks to the cryo-EM resolution evolution and key contributions from the Yan and the Catterall laboratories, several features unique to the mammalian channels were visualized with great clarity (Pan et al., 2018; Pan et al., 2019; Shen et al., 2019; Jiang et al., 2020) (Figure 1C). However, progress on capturing the mammalian channels in different conformations of the functional landscape is still somewhat limited. Hopefully, more advances will be accomplished in the near future.

The α-subunit of human and eukaryotic Na_V_ channels contains >2,000 amino acids that form four homologous but not identical repeats or domains (I-IV) (Figure 1A) (Noda et al., 1984). From the primary sequence, each domain contains six transmembrane helices or segments that form two functionally distinct modules (Catterall et al., 2020a). The first four segments (S1 to S4) form a voltage-sensing domain (VSD) that senses changes in the membrane potential (Figures 1A–C). The last two segments (S5 and S6) in each domain create a pore module (Figures 1D–F). Between the VSD and the pore module is the S4-S5 linker that plays a critical role for channel operation (Catterall et al., 2017) (Figure 1B). All four domains are linked together by intracellular loop regions that have some regulatory roles. The most critical linker is the loop connecting domains III and IV, which contains the fast inactivation gate that closes the pore and inactivates the channel after a brief opening (Figures 1A,C). Other loops and the C-terminus contain some phosphorylation sites and protein-protein interaction motifs (Pei et al., 2018). In three dimensions, all four domains come together in a domain swapped arrangement to form an ion-conducting pore at the center with four pore modules lining the pore structure of the channel and four VSDs located at the peripheries (Figure 1C).

Although bacterial Na_V_ channels do not possess the sophisticated regulatory modules found in eukaryotic channels, their core structures are surprisingly highly conserved within Na_V_ channels from all domains of life (Gamal El-Din et al., 2018a). Bacterial Na_V_ channels are similar to their eukaryotic counterparts in that they also contain six transmembrane segments. However, bacterial Na_V_ channels are expressed as a smaller polypeptide unit containing a single domain (Figure 1B), and use four identical subunits to form a symmetric homotetramer, which operate in a concerted manner. In contrast, each domain in eukaryotic channels is slightly different from one another, creating subtle varieties for intricate operation. For example, each VSD moves in response to different voltage thresholds and controls the pore opening with different kinetics (Chanda and Bezanilla, 2002). The selectivity filter in eukaryotic channels is also asymmetric (Figure 1E), thereby allowing for much greater ion specificity for sodium compared to the filter in prokaryotic channels (Gamal El-Din et al., 2018a).

**FIGURE 2:**
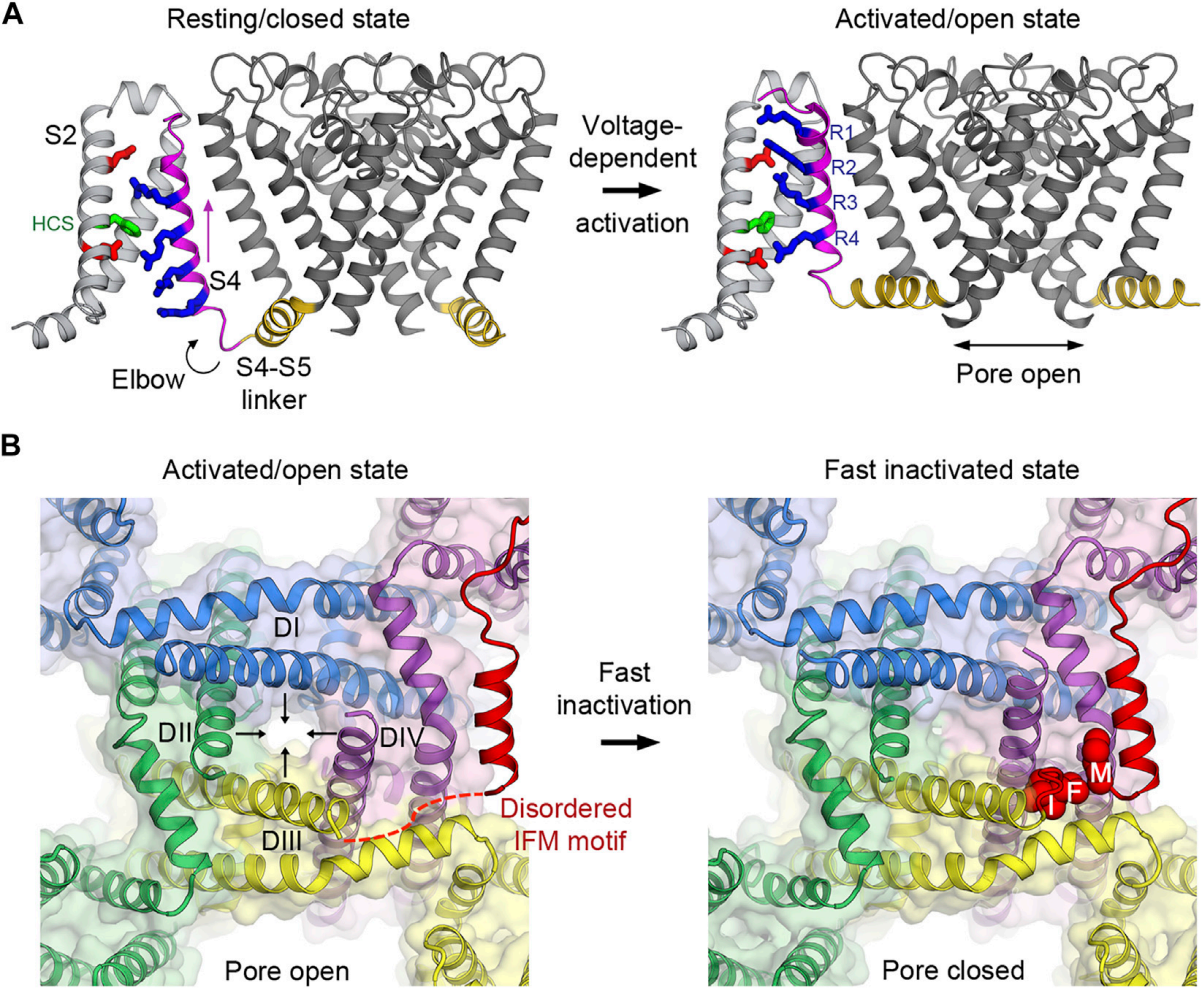
Voltage-dependent activation and inactivation of Na_V_ channels. **(A)** Structural transition of the voltage sensor and the pore during the voltage-dependent activation from resting/closed to activated/open states in the bacterial Na_V_Ab channel. Four gating charge arginines (R1–R4) are shown in blue. Extracellular and intracellular negative clusters are shown in red. Phenylalanine in the hydrophobic constriction site (HCS) is shown in green. S4 (magenta) moves outward, passing two gating charges through the HCS. The S4–S5 linker (gold) connects the S4 movement to gating of the pore. **(B)** Structural transition of the activation gate during the voltage-dependent inactivation from activated/open to fast inactivated/closed states in rat Na_V_1.5. Arrows indicate the directions of movement of the S6 segments. The IFM motif latches onto its receptor site formed by the S4-S5 linker and S6 of DIII and DIV to inactivate the channel and close the pore.

For simplicity, the functional states of Na_V_ channels can be classified into three major conformations: resting/closed, activated/open, and inactivated/closed, with different parts and modules in the structure adopting unique conformations required for each functional step (Figure 2). Here we briefly discuss our current understanding of key processes originally described by Hodgkin and Huxley (Hodgkin and Huxley, 1952a; Hodgkin and Huxley, 1952b; Hodgkin and Huxley, 1952c; Hodgkin and Huxley, 1952d; Hodgkin and Huxley, 1952e; Hodgkin et al., 1952) that facilitate the transitions of Na_V_ channels from one conformation to the next in light of recent molecular details from high-resolution structural studies.

### Voltage-Dependent Activation

From early voltage-clamp experiments on the squid giant axon, Hodgkin and Huxley predicted that voltage sensing involves the movement of the **m** and the **h** charged particles across the membrane electric field during channel activation and subsequent inactivation, respectively (Hodgkin and Huxley, 1952e). The gating currents produced from the movement of these charged particles were later measured in the 1970s (Armstrong and Bezanilla, 1973; Keynes and Rojas, 1973) and recently confirmed by structures of purified Na_V_ channels at high resolution (Payandeh et al., 2011; Shen et al., 2017). These charged particles are now known as the S4 segments. Because the S4 segments contain an array of positive gating charges (arginine and lysine) at every third position (usually 4 to 6 charges per VSD), a negative electrostatic force near the inner membrane at the resting membrane potential pulls the S4 segments toward the cytoplasm. The other three segments (S1 to S3) of the VSDs contain key conserved residues with a negatively charged (glutamate and aspartate) or amide (glutamine and asparagine) side chain known as the extracellular and the intracellular negative charge clusters (ENC and INC, respectively). These residues were proposed to exchange ion pairs with the gating charge residues as the S4 segment progressively translocate from the inward resting-state position outward toward the activated conformations upon membrane depolarization (Catterall, 1986; Guy and Seetharamulu, 1986). A set of hydrophobic residues known as the hydrophobic constriction site (HCS) is located between the ENC and the INC. The HCS serves as a seal that prevents ionic leak through the VSDs and is conserved among voltage sensors from K_V_, Na_V_, Ca_V_, proton channels, and voltage-dependent phosphatase (VSP) enzymes (Tao et al., 2010; Payandeh et al., 2011; Li et al., 2014; Wu et al., 2016). Missense mutations of the gating charge arginines on S4 cause a leak current through the VSD, which is known as the gating pore current (Sokolov et al., 2005; Tombola et al., 2005; Gamal El-Din et al., 2010; Gamal El-Din et al., 2021). The similarity of key functional residues in the voltage sensors of various ion channels from prokaryotes to eukaryotes indicates that the fundamental voltage sensing mechanism has been conserved throughout the evolution.

Most of the voltage-gated ion channel structures determined so far are in the activated or the inactivated states. Without the negative membrane potential in the living cells that stabilizes the resting/closed state, VSDs in the purified Na_V_ channels adopted the activated conformation (with the pore either open (Lenaeus et al., 2017; Sula et al., 2017) or closed (Payandeh et al., 2011; Zhang X. et al., 2012)). To overcome the membrane potential limitation, voltage-shifting mutations and cysteine crosslinking were employed for structure determination of the bacterial homologue Na_V_Ab to capture the resting/closed state (Figure 2A) (Gamal El-Din et al., 2013; Wisedchaisri et al., 2019). Other manipulations using resting state-specific neurotoxins that bind to a VSD resulted in comparable VSD conformations (Xu et al., 2019; Wisedchaisri et al., 2021). Remarkably, similar findings were also observed in other ion channel structures, including the plant AtTPC1 and the mammalian MmTPC1 tandem pore channels (Guo et al., 2016; Kintzer and Stroud, 2016; She et al., 2018) and the human HCN1 hyperpolarization-activated cyclic nucleotide-gated channel (Lee and MacKinnon, 2017, 2019). Together a consensus mechanism that closely follows the sliding helix model for the S4 segment first proposed in the 1980s (Catterall, 1986; Guy and Seetharamulu, 1986) emerges across different types of channels for the movement of the gating charges in the VSDs during activation.

At the resting membrane potential, VSDs in Na_V_ channels adopt the resting conformation determined by the “down” position of the S4 segments which leads to pore closure and the overall channel being in the resting/closed state (Figure 2A). The S4-S5 linkers, which connect S4 of VSDs to the pore modules, form a collar and adopt a restricted conformation that prevents the activation gate from opening as observed in the bacterial Na_V_Ab structure in the resting state (Wisedchaisri et al., 2019). When the membrane potential is depolarized, the charges near the inner side of the plasma membrane are reversed toward positive and repel positive gating charge arginines, causing S4 to move “up” toward the extracellular side of the membrane (Bezanilla, 2008). It has been estimated that 6–14 elementary charges cross the focused electric field during this outward movement of S4 in the VSDs (Hirschberg et al., 1995; Gamal El-Din et al., 2008). The movement of S4 pulls the S4-S5 linker like an elbow to loosen the tight collar around the C-terminus of S6 segments, thus allowing S6 to adopt a new conformation that dilates the pore diameter at the intracellular activation gate to become open for Na^+^ influx (Wisedchaisri et al., 2019) (Figure 2A). Therefore, Na_V_ channels operate as electromechanical coupling devices. The VSDs sense changes in membrane potentials and mechanically control the latch made of the S4-S5 linker collar, which in turn governs the pore S6 diameter at the intracellular activation gate. Bacterial Na_V_ channels use this mechanism in all four domains to activate and open the channels (concerted movement) due to their homotetrameric nature.

In mammalian Na_V_ channels, fluorescent labeling studies have indicated that the S4 movement in the VSDs of domains I to III (VSD1 to VSD3) activates the channel and triggers pore opening at the activation gate (Chanda and Bezanilla, 2002). The VSD of domain IV (VSD4) is the last to be fully activated after the pore has opened and its movement subsequently triggers fast inactivation that closes the pore (discussed below). The slow activation of VSD4 is due to two amino acids (one located in the middle of S2, and the other adjacent to the first gating charge on S4) that are hydrophobic (isoleucine and valine) in VSD4, but are hydrophilic (threonine and serine) in the rapidly activated VSD1 to VSD3. These residues act as a speed damper of the VSD activation for each domain and are conserved in all Na_V_ subtypes. It is unclear how far the S4 segment in VSD4 moves upward from its resting state during the rising phase of a depolarization before it becomes fully activated near the peak current. Based on VSD4-specific neurotoxin studies, the toxins trap the S4 segment in an intermediate (partially down by two gating charges) position compared to the inactivated state to stabilize the opened pore and prevent fast inactivation (Clairfeuille et al., 2019; Jiang et al., 2021b).

### Pore Opening and Sodium Selectivity

The pore modules from all four domains form an ion permeation pathway that extends from the extracellular side of the membrane to the intracellular side (Figure 1D). Mammalian Na_V_ channels have an asymmetric selectivity filter composed of the DEKA motif, to which each pore domain (I-IV, respectively) contributes one amino acid (Figure 1E). (The selectivity filter in bacterial Na_V_ channels is four-fold symmetric with four negatively charged glutamate residues forming a high-field-strength site. Side chains of these glutamates temporarily bind and dunk each hydrated Na^+^ ion through the narrow passage of the selectivity filter (Chakrabarti et al., 2013; Catterall et al., 2020a).) Negatively charged residues aspartate (D) and glutamate (E) from domains I and II of mammalian Na_V_ channels probably retain their high-field-strength function over the course of evolution while the lysine (K) and alanine (A) residues from domains III and IV were evolved in eukaryotes with a nervous system to add magnitudes of selectivity for Na^+^ over Ca^2+^ (Gamal El-Din et al., 2018a). The positively charged lysine residue plays a critical role in Na^+^ selectivity but it is unclear how it achieves that function (Favre et al., 1996). Perhaps it acts as a guard that repels the two positive charges of Ca^2+^, but tolerates one positive charge of Na^+^ due to a net charge of zero after combining with D, E, and A. Additionally, Ca^2+^ have a stronger preference for ligand coordination with carboxylate and amide side chains rather than amine in lysine (Zheng et al., 2008). Surprisingly, evolutionary analysis of distantly related sodium leak NALCN channels showed that their selectivity filter changed from EEEE (Ca^2+^ selective) to EEKE (Na^+^ selective) when eukaryotes evolved to have the nervous system, which supports the importance of lysine for sodium selectivity (Senatore et al., 2013). Ongoing computational modeling and simulations are being carried out to address the mechanistic details of the role of Lys in Na_V_ channels (Flood et al., 2018; Zhang et al., 2018).

On the intracellular side of the ion permeation pathway is the intracellular activation gate that constricts the pore diameter for sodium exiting the channel (Figure 1F). Combinations of conformational changes both in the VSD that contact the pore S5 following the upward movement of S4, and in the S4-S5 linker that interacts with S6, are two factors that trigger the dilation of the S6 intracellular activation gate during the electromechanical coupling. Recent structural evidence from bacterial Na_V_ channels has suggested that some S6 helices can adopt different conformations by altering helical hydrogen bond pattern, thus creating a hinge that produces small degrees of rotation enough to turn hydrophobic residues at the intracellular activation gate away from the center of the pore axis and dilate the activation gate diameter to ∼10 Å (McCusker et al., 2012; Lenaeus et al., 2017; Sula et al., 2017; Gamal El-Din et al., 2019). This can happen if there is large enough space created by a loosen collar of the S4-S5 linkers upon voltage sensor activation. However, open pore channels can be toxic if not tightly regulated, as cells need to maintain electrochemical balances. Therefore, the open pore state is generally short lived because of either the channel regulatory components or its own conformational instability.

### Voltage-Dependent Inactivation

The transient open pore is unstable and eventually closes by a process known as inactivation. In mammalian Na_V_ channels, there are two modes of voltage-dependent inactivation: fast and slow (Catterall et al., 2020a). Fast inactivation, first described by Hodgkin and Huxley (Hodgkin and Huxley, 1952e), usually takes 1–5 milliseconds after the channel opens, depending on the channel subtypes, to close the pore. It is a critical step in the channel functional cycle that rapidly terminates each action potential to generate spikes of neuron firings in eukaryotes with the nervous system. The intracellular loop connecting domains III and IV contains the fast inactivation gate (or particle) (Vassilev et al., 1988) with the signature IFM motif followed by a short helix that latches onto its receptor site on the outer side of the pore formed by the S4-S5 linkers of domains III and IV, and the S6 of domain IV (Figure 2B). This receptor site only forms when all the VSDs are activated because the conformations of the S4-S5 linkers are connected to the activation of S4 in the VSD. It has been shown that VSD4 movement is both necessary and sufficient for fast inactivation (Capes et al., 2013). Since the VSDs activate sequentially in both voltage and time due to different voltage sensing thresholds and kinetics, VSD1 and VSD2 are the first to activate, while VSD4 is the last (Chanda and Bezanilla, 2002). Fast inactivation is essentially triggered by the activation of VSD4 that couples the movement of S4 to the S4-S5 linker that forms the receptor site for the IFM motif to allosterically close the pore. The binding of the IFM motif squeezes S6 of domain III and IV by ∼2–∼6 Å, respectively (Jiang et al., 2020; Jiang et al., 2021a), to tilt inward toward the pore axis and close the activation gate (Figure 2B).

Bacterial Na_V_ channels lack the fast inactivation gate and, therefore, do not possess the fast inactivation process (Pavlov et al., 2005; Gamal El-Din et al., 2019). The bacterial S6 segment undergoes two conformational changes; the first one initiates the opening of the activation gate, and the second one begins the process of inactivation. Studies of different bacterial Na_V_ channels revealed that conformational twisting and bending of kink residues in the middle of S6 are required for inactivation during the depolarizing electrical pulse and for early entry into the slow inactivated state (Zhao et al., 2004; Lee et al., 2012; Gamal El-Din et al., 2019). In addition, the long intracellular C-terminal tail unique to bacterial Na_V_ channels mediates the late use-dependent inactivation (Gamal El-Din et al., 2019), of which the functional role is uncertain (Mio et al., 2010; Irie et al., 2012; Arrigoni et al., 2016).

Slow inactivation is a voltage-dependent process that takes a longer time frame to close the pore (Vilin et al., 1999) usually several hundreds of milliseconds to seconds. This mode of inactivation is conserved in both prokaryotic and eukaryotic Na_V_ channels, although the time scales of entry and recovery from it are different (Vilin et al., 1999; Vilin and Ruben, 2001; Irie et al., 2010; Gamal El-Din et al., 2013; Gamal El-Din et al., 2019). It is thought that slow inactivation helps neurons memorize previous excitations (Toib et al., 1998). Mutant Na_V_ channels lacking or disrupting the fast inactivation component (which cause several human diseases due to a prolonged Na^+^ influx) ultimately exhibit slow inactivation after they stay open long enough (Rudy, 1978; Plumereau et al., 2021). Site-directed mutagenesis studies implicated amino acid residues in the selectivity filter and the surrounding area in the S5 and S6 segments in the conformational changes that accompany slow inactivation (Balser et al., 1996; Benitah et al., 1996; Vilin and Ruben, 2001). Mutation of W402 located in the selectivity filter of domain I of rat Na_V_1.4 dramatically reduces slow inactivation (Balser et al., 1996). Cysteine substitution experiments indicated that four charged residues near the selectivity filter of the rat Na_V_1.4 (E403, E758, D1214, and D1532 in domains I to IV, respectively) move toward each other during the establishment of slow inactivation (Xiong et al., 2003) to form disulfide bonds. In addition, accessibility studies indicated that F1236C in the P-loop of domain III was extracellularly accessible to sulfhydryl modification using MTSEA during short depolarizing pulses, but became inaccessible during long depolarizing pulses, which induce slow inactivation (Ong et al., 2000). The structural basis for slow inactivation in mammalian Na_V_ channels is uncertain because all of the available structures so far are likely in the fast-inactivated state which may prevents the channels from undergoing slow inactivation. Slow inactivation is thought to be similar to inactivation observed in some homotetrameric bacterial Na_V_ structures, which involves a collapsed pore in the entire ion permeation pathway. The selectivity filter and the activation gate collapse from four-fold symmetry favorable for sodium coordination to two-fold symmetry that is much narrower in one axis and cannot conduct sodium (Zhang X. et al., 2012; Payandeh et al., 2012). The collapsed pore feature is also used in the C-type inactivation of tetrameric K_V_ channels at the selectivity filter (Hoshi et al., 1991), and has been observed in recent cryo-EM structures of the N-type voltage-gated calcium channel Ca_V_2.2 (Dong et al., 2021; Gao et al., 2021).

### Voltage-Dependent Deactivation

Membrane hyperpolarization following the rising phase of an action potential reverses the membrane potential to a more intracellularly negatively charged and likely pulls S4 in the VSD inward toward the cytoplasm to deactivate the channel (Catterall et al., 2020b). What happens to the selectivity filter, the intracellular activation gate, and the fast inactivation gate during this process and how many steps are involved is largely unknown. We consider the selectivity filter and the activation gate to remain unchanged during the deactivation process following fast inactivation. Perhaps a small shift of S6 at the activation gate occurs while the pore remains closed, and the fast inactivation gate becomes unlatched and disordered as its receptor site is dismantled upon deactivation of the VSDs. Others have speculated a different mechanism from structures of mammalian Ca_V_ and insect Na_V_Pas channels in which a motif analogous to the fast inactivation gate is bound to the intracellular C-terminal domain when not in use (Wu et al., 2016; Shen et al., 2017; Clairfeuille et al., 2019). However, those channels lack both the fast inactivation process and the IFM motif found in the fast inactivation gate of vertebrate Na_V_ channels, casting doubt on the validity of this model. On the other hand, the cycle involving the slow-inactivation process is hard to speculate about. Hopefully this area will become clearer when more structures in the currently unrepresented states are captured.

## Natural Druggability of Voltage-Gated Sodium Channels

**FIGURE 3:**
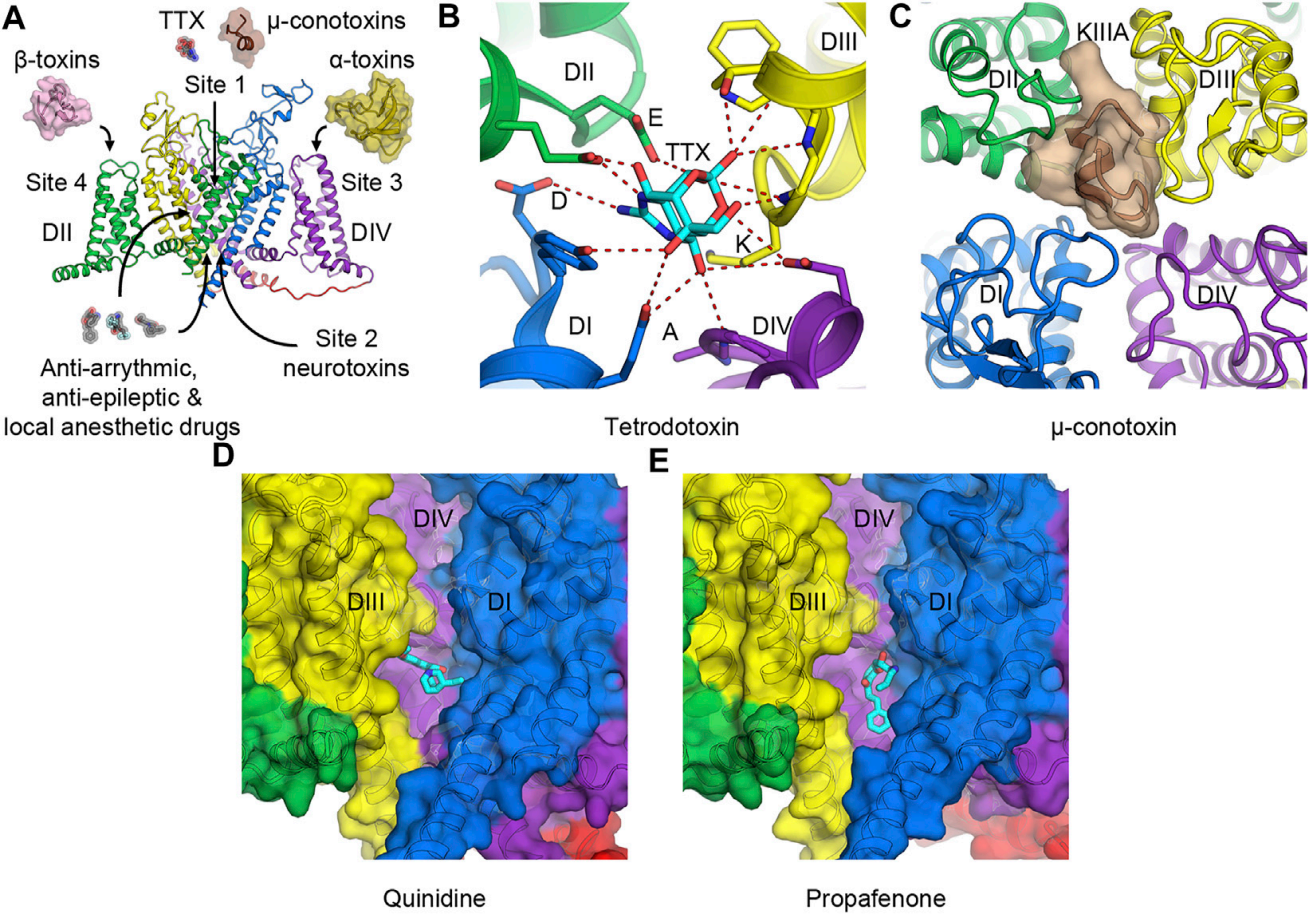
Toxins and drugs that physically block the pore. **(A)** Neurotoxin and drug receptor sites on Na_V_ channels. Receptor sites 1 to 4 are illustrated with their representative natural modulators. Anti-arrhythmic, anti-epileptic, and local anesthetic drugs enter the hydrophobic central cavity in the pore through the membrane fenestration and the activation gate. **(B)** Tetrodotoxin (TTX) binds to receptor site 1 at the selectivity filter and interacts with the DEKA motif of human Na_V_1.7. **(C)** μ-Conotoxin KIIIA binds to receptor site 1 at the extracellular mouth of the channel above the selectivity filter of human Na_V_1.2. **(D,E)** Anti-arrhythmic drug class IA quinidine, **(D)** and class IC propafenone, **(E)** bind to Na_V_1.5 in the central cavity of the pore formed by DI and DIV. For clarity, the pore module of DII has been removed.

Na_V_ channels are targets of numerous naturally occurring plant and animal neurotoxins that exploit several distinct druggable sites on the channels for binding and modulating the channel functions (Figure 3A). They also contain receptor sites for local anesthetics and many clinical drugs for treating human diseases (Noreng et al., 2021). Six neurotoxin receptor sites have been identified and characterized in great detail (Catterall et al., 2007). Recent successes in structure determination at high resolution by cryo-EM allowed unprecedented structural characterization of some of these receptor sites with their associated neurotoxins. Here we provide a brief overview of the recent structurally characterized neurotoxin receptor sites in the pore and the VSDs to demonstrate diversities in druggability of Na_V_ channels. Readers are referred to (Stevens et al., 2011; Noreng et al., 2021) for comprehensive reviews on this topic. These natural druggable sites could serve as a starting point both for drug design of novel classes of compounds to target the same sites, and for optimization of existing drugs or ligands to fine-tune the function of Na_V_ channels.

### Toxins That Bind to the Pore Module

Receptor site 1 is located at the extracellular mouth of the channel right above the selectivity filter (Figures 3A–C). Neurotoxins that act on site 1 include small molecule guanidinium toxins such as tetrodotoxin (TTX) from pufferfish (Figure 3B), and saxitoxin (STX) from marine dinoflagellates and cyanobacteria. They also include small peptides such as μ-conotoxin from cone snails (Figure 3C). These toxins bind to the receptor site formed by portions of the P-loops that connect S5 to S6. They sit above the DEKA motif of the selectivity filter and physically block the pore to prevent Na^+^ entry. As binding of TTX inhibits slow inactivation, certain residues that form the TTX-binding site have been implicated in the slow inactivation process (Capes et al., 2012). Cryo-EM structures of human Na_V_1.7 and cockroach Na_V_Pas with TTX or STX illustrate that the toxins use positive charges of guanidinium group to interact with the negative charged residues in the DEKA motif of the selectivity filter (Shen et al., 2018; Shen et al., 2019) (Figure 3B). A tyrosine adjacent to the aspartate in domain I also forms a π-cation interaction with the guanidinium groups of the toxins that provide a high affinity for the binding in TTX- and STX-sensitive Na_V_ subtypes. This tyrosine residue becomes cysteine (Na_V_1.5) or serine (Na_V_1.8, and Na_V_1.9) in TTX-insensitive channels with much lower affinity for the toxin (Jiang et al., 2020). Similarly, μ-conotoxin KIIIA binds to human Na_V_1.2 at the extracellular opening of the pore on top of the P-loop structure from all four domains and occludes the selectivity filter (Pan et al., 2019) (Figure 3C).

Receptor site 2 is in the central cavity of the pore domain and formed by the internal wall of the S6 segments (Figure 3A). Neurotoxins that bind to this site are small molecule steroidal alkaloids (batrachotoxin and veratridine) and norditerpenoid alkaloids (aconitine and its analogs) that enter the pore when the channel is open. These toxins cause a combination of a hyperpolarization shift in voltage-dependent activation, a prolonged sodium current entry (by preventing fast inactivation), and a reduced peak sodium current (due to partial pore blockage) (O'Leary and Krueger, 1989). A detailed mechanism for this class of toxins is currently unknown due to the lack of high-resolution structures of the toxins bound to Na_V_ channels. Biochemical, functional, and computational modeling data suggest that the toxins wedge between the S6 segments of domains I, III, and IV and likely keep the S6 segments from closing (Correa et al., 1992; Linford et al., 1998; Wang and Wang, 2017; Craig et al., 2020).

### Drugs That Bind in the Central Cavity of the Pore

Local anesthetic, anti-arrhythmic, and anti-epileptic drugs act on Na_V_ channels by binding in the central cavity at the site that partially overlaps with receptor site 2. These drugs enter the central cavity through the intracellular activation gate when the channel is open and through a small tunnel known as a fenestration from the plasma membrane (Gamal El-Din et al., 2018b; Hille, 1977; Ramos and O'Leary, 2004). Class I anti-arrhythmic drugs targeting the cardiac Na_V_ channel have been classified into three subclasses (IA to IC) based on their effects on the ventricular action potential. The complex therapeutic actions of these drugs arise from three different modes of state-dependent block: slow resting-state block, rapid open-state block, and high-affinity inactivated-state block. The state-dependent block allows local anesthetics (LAs) and anti-arrhythmic drugs (AADs) to selectively prevent generations of high-frequency action potentials that are characteristic of intense pain and cardiac arrhythmia, respectively, but assert little effect on normal electrical signaling. Two hypotheses have been proposed to explain the mechanism of LAs and AADs actions. The Modulated Receptor Hypothesis posits that the resting-state block is mediated by drug entry from the lipid phase of the membrane into the drug receptor site in the pore at the resting state, while the rapid open-state block occurs as the drug enters the open pore from the cytoplasm (Hille, 1977). Both modes of block are enhanced when the channel enters the inactivated state, which has higher affinity for the bound drugs. On the other hand, the Guarded Receptor Hypothesis postulates that accessibility of the receptor is the main factor to determine the potency of LAs and AADs (Starmer et al., 1984). In this model, the open conformation of the channel controls the flux of the drugs as they diffuse between drug pools and the binding site. Two residues in domain IV-S6 (F1760 and Y1767 in human Na_V_1.5) have been shown to play a critical role in mediating the use-dependent block of these drugs (Ragsdale et al., 1994). Cryo-EM structures of human and rat Na_V_1.5 with anti-arrhythmic drugs classes IA (quinidine) (Figure 3D) and IC (propafenone) (Figure 3E) confirm that these drugs bind to the domain I-S6 and the domain IV-S6 segments and interact with the conserved F1760 side chain (human Na_V_1.5 numbering) in domain IV-S6 via a π-π interaction (Jiang et al., 2020; Jiang et al., 2021a; Li et al., 2021). The structures also show the drugs interacting with Y1767 as has been suggested by previous electrophysiology and mutagenesis studies (Ragsdale et al., 1996). Class IA and IC anti-arrhythmic drugs are open channel blockers. Class IB drugs (lidocaine) on the other hand bind to the channel in both the open and the inactivated states and block the channel by stabilizing the inactivated/closed state (Bean et al., 1983; Pless et al., 2011). Detailed discussion on molecular determinants of state-dependent drug binding in the central cavity can be found in (O'Leary and Chahine, 2018).

### Toxins That Bind to Voltage-Sensing Domains to Modulate the Pore

Neurotoxins that act on voltage sensors are cysteine knot peptides from spiders, scorpions, and sea anemones. Based on their topology and conservations of cysteines, they are evolutionarily related to defensins (Zhu et al., 2015), which are the host defense peptides from various organisms. Their folds are conserved and their structures are highly stable because of constraints from multiple disulfide bonds. However, their functions can differ significantly due to varying shapes and charges presented on the surface that provide high binding affinity to specific receptor sites on different ion channels and domains. Toxins that bind to VSDs are considered gating modifiers because they modulate Na_V_ channels by trapping the VSDs in a particular state, which modifies the gating of the pore to either keep the pore closed or open.

**FIGURE 4:**
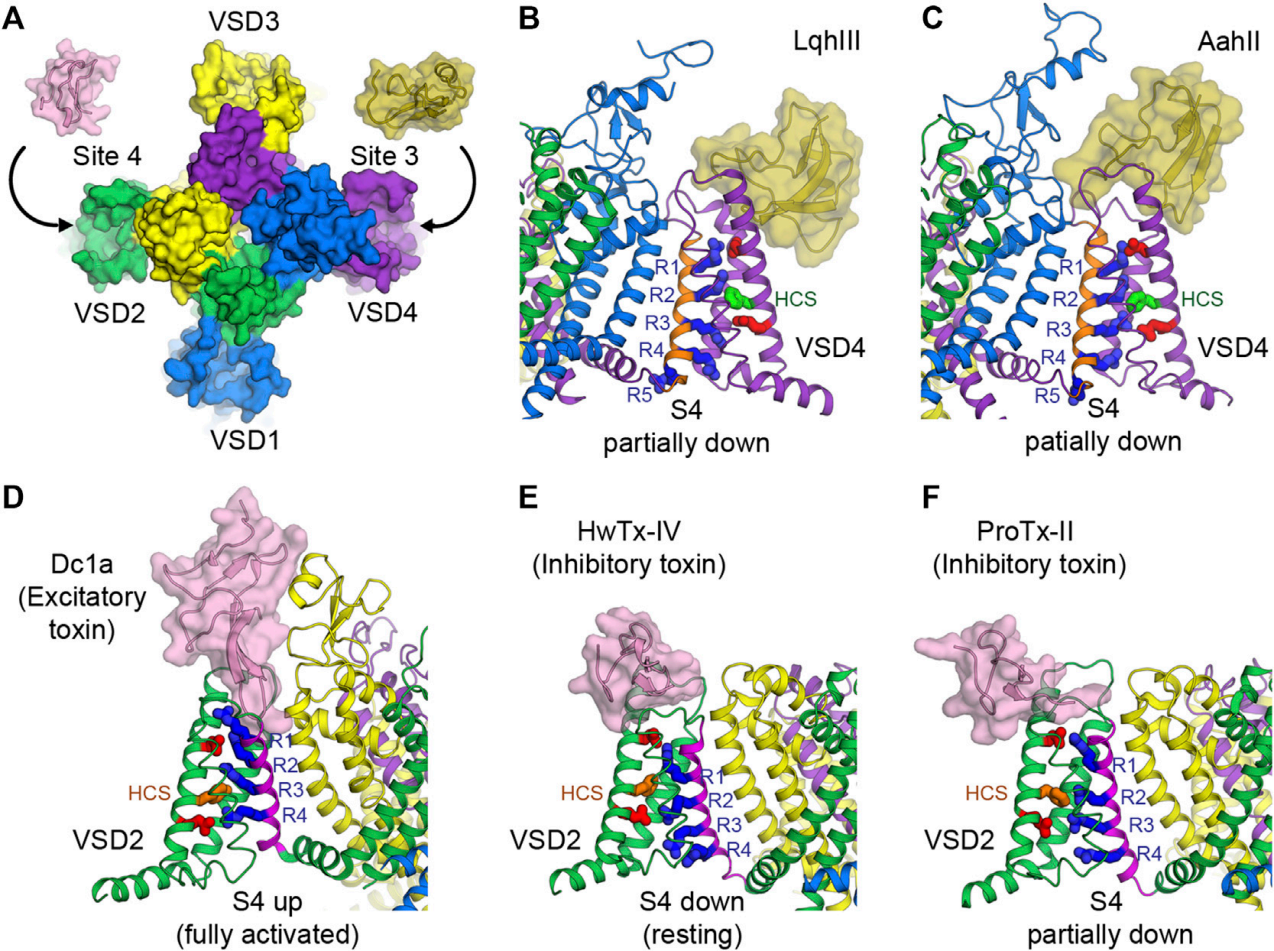
Toxins that bind to voltage sensors to modulate the pore. **(A)** Receptor sites 3 and 4 at the extracellular aqueous clefts of VSD4 and VSD2, respectively, are targets of animal cysteine knot peptides. **(B,C)** Site 3 scorpion α-toxins LqhIII **(B)** and AahII **(C)** bind to VSD4 of rat Na_V_1.5 and cockroach Na_V_Pas/human Na_V_1.7-VSD4 chimera, respectively, to stabilize S4 (orange) in the partially down conformation, as 2 gating charges are external to the HCS. The gating charge arginines (R1–R5) are shown in blue. Extracellular and intracellular negative clusters are shown in red. Phenylalanine in the HCS is shown in green. **(D)** Site 4 excitatory spider toxin Dc1a binds to VSD2 of cockroach NavPas and traps S4 (magenta) in the activated state, as three gating charges are external to the HCS. Phenylalanine in the hydrophobic constriction site (HCS) is shown in orange. **(E,F)** Site 4 inhibitory spider toxins HwTx-IV **(E)** and ProTx-II **(F)** trap VSD2 of Na_V_Ab/human Na_V_1.7-VSD2 chimera in the resting state and the deactivated state, as 1 and 1.5 gating charges are external to the HCS, respectively.

Receptor site 3 is located on the extracellular aqueous cleft of the VSD in domain IV (VSD4) and part of the extracellular portion of the pore (Figures 3A, 4A). Site 3 toxins are α-toxin peptides produced by old world scorpions and sea anemone that selectively bind to a specific Na_V_ subtype with a very high affinity, usually in the range of nanomolars. Since VSD4 is responsible for triggering fast inactivation, site 3 toxins that bind and trap VSD4 in the resting (or the intermediate) state prevent the channel from undergoing fast inactivation and cause it to stay open. Cryo-EM structures of rat Na_V_1.5 and coackroach Na_V_Pas/human Na_V_1.7-VSD4 chimera with scorpion α-toxins LqhIII (Figure 4B) and AahII (Figure 4C), respectively, reveal a consensus mechanism of α-toxins action. By stabilizing VSD4-S4 in the “down” conformation (by two gating charges compared to the activated VSD4), the toxins ultimately prevent the channels from undergoing fast inactivation (Clairfeuille et al., 2019; Jiang et al., 2021b).

Similar to site 3, receptor site 4 is on the extracellular aqueous cleft of the VSD in domain II (VSD2) (Figures 3A, 4A), with a major contribution from the S3-S4 loop, which is known to be the gating-modifier hot spot. Interestingly, this hot spot is well exploited and utilized by various gating modifier toxins that target different ion channels (Catterall et al., 2007; Winterfield and Swartz, 2000). However, for some larger toxins such as scorpion toxins, the binding may include part of the extracellular portion of the pore. Neurotoxins targeting this site are β-toxin peptides from new world scorpions and spiders. These neurotoxins can be grouped into 2 major functional classes based on their outcomes: excitatory and inhibitory. Excitatory toxins bind VSD2 in the activated state and enhance channel activation. Inhibitory toxins, on the other hand, bind VSD2 in the resting state and prevent channel from activation. Cryo-EM structure of cockroach Na_V_Pas with Dc1a toxin from a spider demonstrates the binding of excitatory toxins that trap VSD2 in the activated state (Shen et al., 2018) (Figure 4D). Recent cryo-EM structures of Na_V_Ab/human Na_V_1.7-VSD2 chimera with Huwentoxin-IV (HwTx-IV) (Figure 4E) and Protoxin-II (ProTx-II) (Figure 4F) from spiders illustrate the binding of inhibitory toxins that trap VSD2 in the resting and deactivated states, respectively (Xu et al., 2019; Wisedchaisri et al., 2021). In the cellular context, toxins can sample the conformational landscape of the channel located in the membrane and stabilized by the resting membrane potential. However, during protein purification carried out at 0 mV potential, channels would become activated or inactivated, making it difficult to trap VSD2 in the resting state even for toxins with high binding affinity. In the case of HwTx-IV, which binds to human Na_V_1.7 VSD2 at nanomolar affinity, a Na_V_Ab/Na_V_1.7-VSD2 chimera with the largest positive shift in the voltage dependence of activation was selected for cryo-EM study. This was crucial for complex formation in the resting state where S4 moves downward by two gating charges from the activated state (Wisedchaisri et al., 2021). Binding of the toxin to unmodified human Na_V_1.7 and NachBac/Na_V_1.7-VSD2 chimeric channels led to the complexes with VSD2 adopting activated conformations (Shen et al., 2019; Gao et al., 2020). In the case of ProTx-II, two populations were present in the same sample, one being VSD2 fully activated and the other (∼18%) being VSD2 deactivated (S4 moves down by 1.5 gating charges) (Xu et al., 2019). So far, high-resolution structural characterization of site 4 neurotoxins is limited to spider toxins, and no structure with scorpion β-toxins have been successfully reported. It is likely that scorpion β-toxins such as CssIV, which are larger than spider toxins, require additional binding from the pore domain similar to site 3 toxins (Zhang JZ. et al., 2012), and thus require a full eukaryotic channel that is more demanding to study compared to the chimeric approach with bacterial channels.

## Innovative Druggability of Voltage-Gated Sodium Channels

Na_V_ drugs that have been approved by the FDA and are being used in the clinics are pore blockers that target the pore domain and occlude the sodium permeation pathway to stop sodium influx (Gamal El-Din et al., 2018b; Jiang et al., 2020; Jiang et al., 2021a; Li et al., 2021). These are local anesthetic (i.e., lidocaine, benzocaine, etc.), anti-arrhythmic (i.e., quinidine, flecainide, etc.), and anti-epileptic (i.e., phenytoin, carbamazepine, lamotrigine, lacosamide, etc.) drugs. Several experimental drugs that target Na_V_1.7 have been developed and gone through clinical trials over the last decade with the goal of treating neuropathic pain (de Lera Ruiz and Kraus, 2015; Kingwell, 2019; Alsaloum et al., 2020). However, none of these drugs have received FDA approval so far. Some of these compounds are pore blockers (e.g., vixotrigine) that target the central cavity. Because of the relatively high homology across the Na_V_ subtypes in the central pore, achieving subtype selectivity for drugs that target this area is still quite challenging.

### Druggability of the P-Loops

There have been some current interests in drug development based on site 1 neurotoxins or their analogues to target the P-loop and the selectivity filter of Na_V_1.7 as pain therapeutics. TTX at a non-lethal concentration has shown prominent analgesic effects in the rodent pain models (Gonzalez-Cano et al., 2021). WEX Pharmaceuticals have recently completed phase 3 clinical trials using TTX for treatment of cancer-related pain and chemotherapy-induced peripheral neuropathy (CIPN) (Hagen et al., 2017; Goldlust et al., 2021). Recently, SiteOne Therapeutics has developed derivatives of STX that exhibit great selectivity toward Na_V_1.7 (Pajouhesh et al., 2020) due to two amino acids that are unique to Na_V_1.7 in the P-loop of domain III (Thomas-Tran and Du Bois, 2016). These bis-guanidinium compounds blocks Na_V_1.7 in a state independent manner and have shown great efficacy in the mouse models of pain (Beckley et al., 2021). SiteOne Therapeutics is currently completing phase 1 clinical trials with compound ST-2427. Phase 2 clinical trials for acute post-operative pain are reportedly underway.

### Druggability of the Voltage-Sensing Domains

Recently, a new approach of developing drugs that target VSDs have gained some momentum. This is because, unlike the pore, VSDs in different Na_V_ subtypes are much more distinct from each other. Therefore, obtaining highly selective drugs toward a specific Na_V_ subtype can be achieved in principle. In addition, VSDs provide unique state-dependent receptor sites that can be explored for drug development. As discussed earlier, VSDs can adopt either the resting, the activated, or the inactivated state (and perhaps additional intermediate conformations that are not currently well characterized by structural biology tools), thereby providing opportunities for drugs that target a specific state to be developed to allosterically modulate the pore. The most useful states of Na_V_ to inhibit are the states in which the activation gate is closed. Drugs that lock VSDs in either the resting state or the inactivated state will facilitate or stabilize the closed pore conformation and inhibit the channel conductance.

### Targeting the Resting State

**FIGURE 5:**
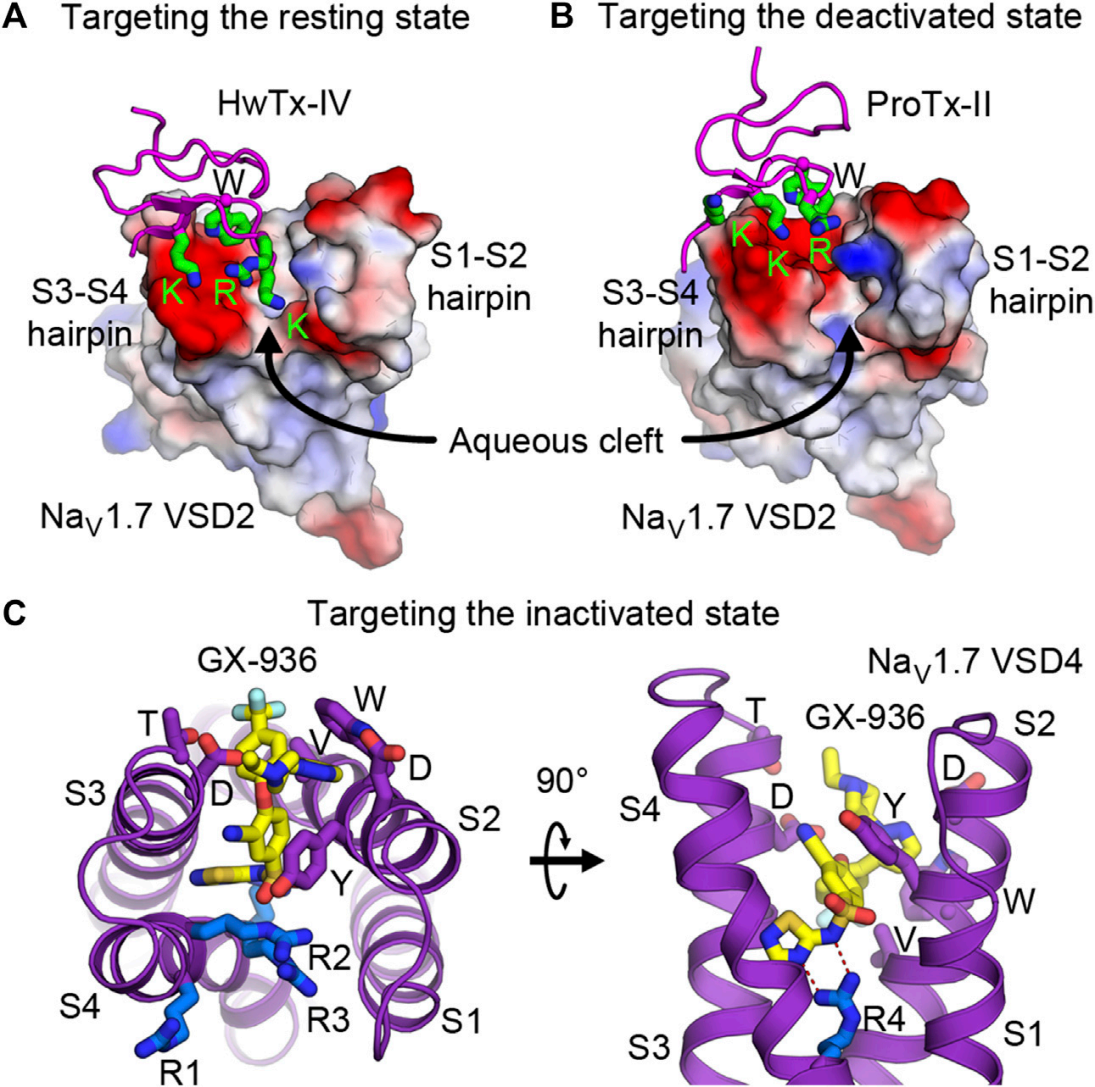
Druggability of the voltage sensor in the resting, deactivated, and inactivated states. **(A,B)** Structures of HwTx-IV **(A)** and ProTx-II **(B)** bound to the aqueous cleft of Na_V_Ab/human Na_V_1.7-VSD2 chimera. Molecular surface of VSD2 is colored by electrostatic potentials (red—negative; blue—positive). Positively charged lysine and arginine residues of the toxins interact with negatively charged residues located in the S3-S4 hairpin. A tryptophan residue from the toxins provides additional van der Waals interaction with the receptor. **(C)** GX-936 aryl sulfonamide antagonist bound to the aqueous cleft of Na_V_Ab/human Na_V_1.7-VSD4 chimera. Carbon atoms of GX-936 are colored in yellow. Residues from VSD4 forming the binding site are shown as sticks. The gating charge arginines R1-R4 are shown in blue.

Development of drugs that target the resting state is in its infancy. This is partly because little structural information has been known previously of the VSDs in the resting state. However, nature has given us plentiful examples of spider and scorpion toxin peptides (i.e., site 4 toxins) that inhibit Na_V_ by trapping VSD2 in the resting state. Numerous discoveries have identified and characterized natural toxin peptides from these groups of animals that show high affinity and selectivity toward Na_V_1.7. Currently, several studies have engineered and optimized a handful of natural toxins into high affinity ligands that are highly selective toward Na_V_1.7 (Adams et al., 2022; Neff et al., 2020; Shcherbatko et al., 2016; Wu et al., 2018). Janssen Pharmaceuticals recently developed and characterized an engineered ProTx-II analog (JNJ63955918) that showed great specificity toward Na_V_1.7 and promising efficacy for treating pain in a mouse model (Flinspach et al., 2017). With recent structural information from cryo-EM studies of Na_V_Ab/Na_V_1.7-VSD2 chimera in the resting and the deactivated states with bound HwTx-IV (Figure 4E) and ProTx-II (Figure 4F), respectively (Wisedchaisri et al., 2019; Xu et al., 2019), development of small molecule ligands specific for VSD2 in the resting state is likely to become more practical. These toxins bind to the S3-S4 loop and occupy part of the aqueous cleft on the extracellular side created by the S1-S2 and S3-S4 hairpins (Figures 5A,B). The shape of the VSD2 aqueous cleft is quite different between the resting and the activated states due to different conformations of the S3-S4 hairpin depending on the position of S4 (resting state = S4 down, activated state = S4 up). Small molecules that mimic the toxins and bind in the aqueous cleft of the VSD in domains I or II with high affinity could potentially trap the VSD in the resting state and thus keep the channel in the closed state.

### Targeting the Inactivated State

Efforts to develop drugs targeting VSDs in the inactivated state have been quite prolific. Early success from Pfizer in developing aryl sulfonamide compounds ICA-121431 and PF-04856264 that have nanomolar affinity and selectivity towards Na_V_1.3 and Na_V_1.7, respectively (McCormack et al., 2013) paved the way for additional compounds from the same class to be developed by several pharmaceutical companies. Based on electrophysiological studies, these compounds bind to the channels in VSD4 in the inactivated state and inhibit the channels from reopening, with residues from S2 and S3 as key determinants for isoform selectivity. Mechanism of action for this class of aryl sulfonamides was later elucidated in great detail by joint ventures from Genentech and Xenon Pharmaceuticals (Ahuja et al., 2015) to develop pain therapeutics for Na_V_1.7. Compound GX-936 and related inhibitors investigated in the study utilize their negatively charged aryl sulfonamide warhead to form a salt bridge with the fourth gating charge arginine (R4) on S4 and stabilize VSD4 in the activated conformation (Figures 5C,D). These compounds essentially inhibit Na_V_1.7 through a voltage-sensor trapping mechanism, by stabilizing the inactivated state of the channel after S4 of VSD4 moves up to trigger fast inactivation. Their binding prevents channel deactivation, thus reducing channel availability to reopen during a train of action potentials. Despite extensive research on developing aryl sulfonamide analogs as novel analgesics, only the compound PF-05089771 from Pfizer eventually entered phase II clinical trials for wisdom tooth removal, osteoarthritis of the knee, primary erythromelalgia, and painful diabetic peripheral neuropathy (McDonnell et al., 2018). Genentech and Xenon Pharmaceuticals have two compounds (GDC-0276 and GDC-0310) that completed phase I clinical trials (Rothenberg et al., 2019; Safina et al., 2021). Further clinical development of these compounds appears to be discontinued.

## Perspective on Novel Druggable Sites on VOLTAGE‐GATED SODIUM Channels

**FIGURE 6:**
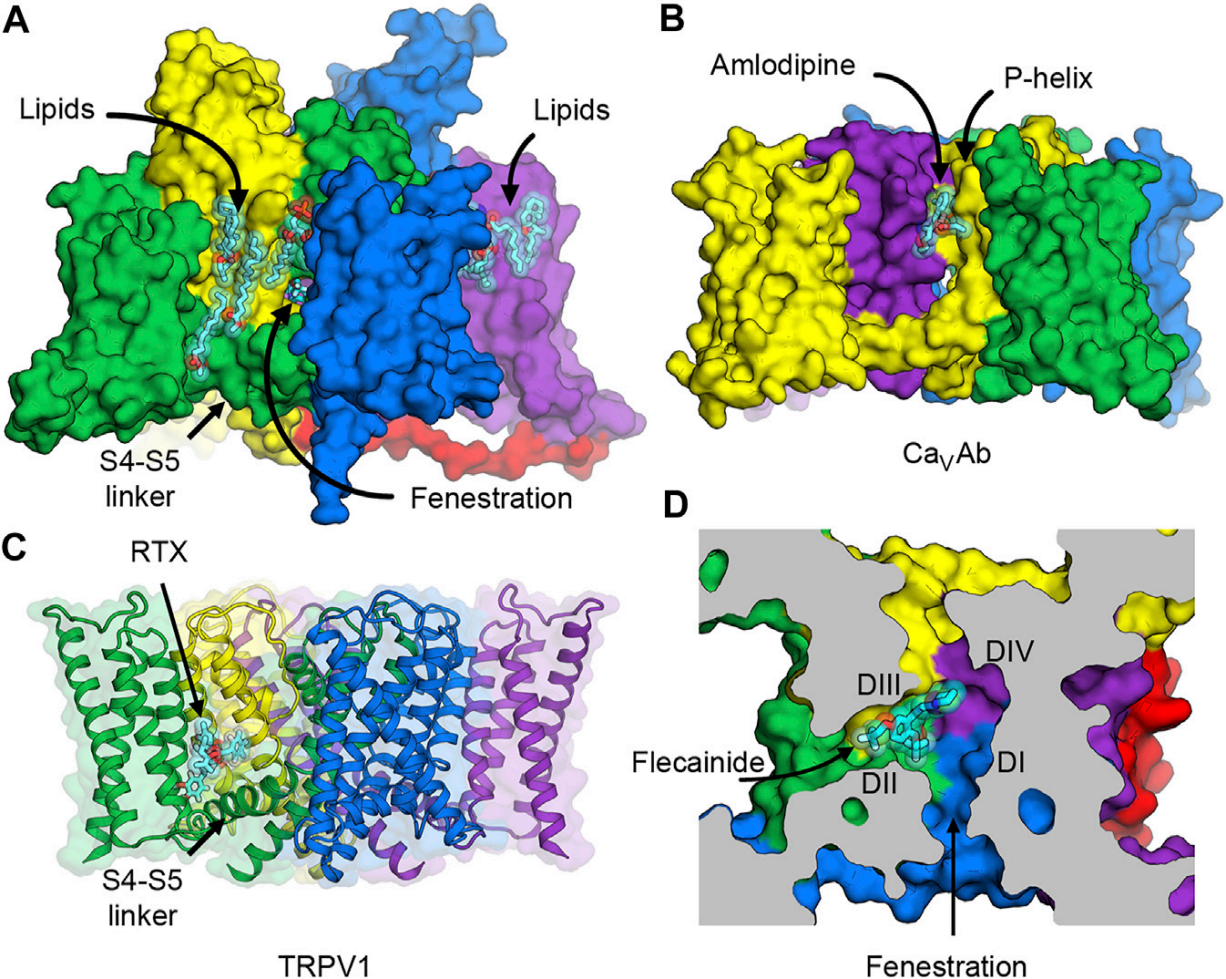
Putative druggable sites on Na_V_ channels. **(A)** The hydrophobic surface between the VSD and the pore of rat Na_V_1.5 channel interacts with lipids in the membrane. Lipid and ligand molecules are illustrated as cyan sticks overlaid with semi-transparent van der Waals spheres. Class IC anti-arrhythmic drug flecainide can be seen through the fenestration window into the pore. Tight binding of lipids suggests possible druggable sites on the lipid face of Na_V_ channels. **(B)** Putative druggable site at the level of the outer membrane leaflet near the P-loop. Amlodipine DHP binds to engineered calcium channel Ca_V_Ab at the inter-subunit crevice formed by neighboring S6 helices and the P-helix to trigger allosteric changes at the selectivity filter. **(C)** Putative druggable site at the level of the inner membrane leaflet near the S4-S5 linker. Resiniferatoxin (RTX) binds in the vanilloid pocket formed by residues from S3 and S4 in the VSD, the S4-S5 linker, and S5 and S6 of the pore, and displace resident phospholipid in this pocket to activate TRPV1 channel. **(D)** Putative druggable site in the fenestration. Flecainide binds in the fenestration of rat Na_V_1.5 formed between DII and DIII of the pore.

Besides the conventional neurotoxin receptor sites on Na_V_1.7 that are being explored for drug development of novel analgesics, new voltage-dependent druggable sites can provide additional opportunities for designing subtype selective ligands. Three possibilities come to our mind. The first two occur on the lipid face of the channel outside the pore between one of the VSDs and the pore of a neighboring subunit (Figure 6A). The other is in the fenestration—a small tunnel that provides access for hydrophobic drugs to enter the central cavity of the pore from the plasma membrane.

### Putative Druggable Sites on the Lipid Face of the Pore

The hydrophobic area on the outer surface of the pore of Na_V_ channels normally interacts with lipids in the membrane. Phospholipid molecules have been observed in several structures of Na_V_ channels that were determined both with added lipids in the preparation and without but carried over during protein purification due to high biding affinity (Figure 6A). Currently there is no consensus evidence that supports the regulatory role of particular lipids in Na_V_ channels, but protein-lipid interactions are hallmarks of membrane proteins and channels that require further investigation.

The first potential druggable site is on the extracellular half of the lipid face at the level of the outer membrane leaflet and the P-loop (Figures 6A,B). In related L-type voltage-gated calcium channels (Ca_V_), this area is the target of dihydropyridine (DHP) calcium channel blockers that are used to treat high blood pressure and severe angina (Figure 6B). The structure of an engineered bacterial calcium channel Ca_V_Ab (Tang et al., 2014) in complex with amlodipine revealed the DHP binding site on the outer, lipid-facing surface of the pore module in the inter-subunit crevice formed by neighboring S6 helices and the P-helix of the selectivity filter (Tang et al., 2016). Subsequently, the structure of rabbit Ca_V_1.1 bound to nifedipine validated the DHP biding site in a mammalian L-type Ca_V_ channel (Zhao et al., 2019a). DHP binding triggers allosteric changes at the selectivity filter of the L-type Ca_V_ channels, and thus alters the binding of calcium ion by disturbing the hydration shell of calcium ions that must be transmitted in a hydrated form through the selectivity filter (Tang et al., 2016). Because sodium ions also permeate through the selectivity filter of Na_V_ channels in a hydrated form, designing a new set of drugs that target the hydrophobic site equivalent to the DHP binding site could potentially lead to an inhibition of sodium channels in the same way that DHPs inhibit calcium flux in L-type Ca_V_ channels.

The second putative druggable site is located on the intracellular half of the lipid face at the level of the inner membrane leaflet between the VSD and the S4-S5 linker (Figures 6A,C). Several structures of Na_V_ channels from bacteria to human indicate a common preference for phospholipids at this site. Therefore, small molecule ligands with high affinity may have a potential to displace lipids and stabilize the VSD and/or the S4-S5 linker in a certain conformation and trap the channel in a state-dependent manner. Interestingly, this hydrophobic surface overlaps with the binding site for pyrethroid insecticides found in insect Na_V_ channels (neurotoxin site 7) (Field et al., 2017), and the vanilloid receptor site in transient receptor potential vanilloid (TRPV) channels. Pyrethroid insecticides act on insect Na_V_ channels by stabilizing the open state of the channels and inhibiting the channel deactivation that results in excitatory paralysis and death (Bradberry et al., 2005; Tan et al., 2005). Currently, structures of Na_V_ channels with bound pyrethroids have not been determined, but several mutagenesis and computational modeling studies have suggested that the S4-S5 linker and the pore S5 and S6 segments form the pyrethroid-binding site (Du et al., 2013; Du et al., 2016; Zhorov and Dong, 2017). Another overlapping binding site is well characterized in TRPV channels, which are distantly related to Na_V_ channels. In TRPV channels, vanilloid ligands such as capsaicin and resiniferatoxin (RTX) bind to the vanilloid pocket formed by residues from S3 and S4 in the VSD, the S4-S5 linker, and S5 and S6 of the pore, and displace a phopsphatidylinositol lipid that normally occupies this pocket in the closed state to activate the channel (Gao et al., 2016; Zhang et al., 2021) (Figure 6C). Recently, synthetic peptides mimicking the S4-S5 linkers have been shown to stabilize the open state of human Na_V_1.4 (Malak et al., 2020), highlighting an innovative strategy for ligand design that modulate the pore by targeting the electromechanical coupling of the channel.

So far, ligands that bind to the lipid face near the S4-S5 linkers and stabilize the resting/closed state of Na_V_ have not been identified. Both pyrethroids and vanilloids are well known agonists that bind in this area in their respective channels and activate the insect Na_V_ and the TRPV channels, respectively. Novel ligands that bind to this receptor site in Na_V_ channels in the resting state or the inactivated state may stabilize the channel in the closed state and prove to be useful inhibitors for potential drug development. They will need to be at least neutral at the physiological pH in order to partition into the membrane and have high enough affinity to displace resident lipids and lock the S4-S5 linker from changing the conformation caused by membrane depolarization/hyperpolarization that activates/deactivates the channels through S4 movement in the VSD. Interestingly, using a drug screening approach, a class of tautomer compounds has been found to specifically open K_V_1-type Shaker and K_V_1.5 potassium channels, presumably by docking into the bottom of the VSD and the S4-S5 linker to stabilize the open channels (Silvera Ejneby et al., 2020). Such an analogous approach could be used to screen for compounds that inhibit Na_V_ channels.

### Putative Druggable Site in the Fenestration

The third druggable site is the fenestration (Figures 6A,D). The existence of the lateral hydrophobic portals as a drug access pathway had been speculated for over 40 years ago (Hille, 1977) until 2011, when the first crystal structure of bacterial Na_V_Ab revealed four fenestrations connecting the lipid phase of the membrane to the central cavity of the channel (Payandeh et al., 2011). Over the past decades, fenestrations were considered merely as the hydrophobic pathway for neutral drugs to enter the pore from the membrane (Gamal El-Din et al., 2018b). However, many recent structures of voltage-gated sodium and calcium channels provide evidence that some drugs have their binding sites that are partially or completely inside the fenestrations (Figure 6D) (Zhao et al., 2019a; Zhao et al., 2019b; Jiang et al., 2020; Dong et al., 2021). Since some residues in the P-loop helices form the roof of the fenestrations, drug binding in the fenestrations may elicit conformational changes of these amino acids and induce a collapse of the selectivity filter to prevent ion permeation.

Obviously, it is unclear how useful these novel sites will turn out to be for drug discovery. However, in our opinion, they are valuable as alternatives that maybe worth exploring from a structure-based drug design perspective.
